# Adaptation of the barriers to help-seeking for trauma (BHS-TR) scale: a cross-cultural cognitive interview study with female intimate partner violence survivors in Iceland

**DOI:** 10.1186/s41687-021-00295-0

**Published:** 2021-02-27

**Authors:** Karen Birna Thorvaldsdottir, Sigridur Halldorsdottir, Rhonda M. Johnson, Sigrun Sigurdardottir, Denise Saint Arnault

**Affiliations:** 1 School of Health Sciences, University of Akureyri, Akureyri, Iceland; 2 Department of Health Sciences, University of Alaska Anchorage, Anchorage, AK USA; 3 School of Nursing, University of Michigan, Ann Arbor, MI USA

**Keywords:** Translation, Cross-cultural adaptation, Cognitive interviews, Self-reported measures, Trauma, Help-seeking, Interpersonal violence, Health-related quality of life

## Abstract

**Background:**

Even though traumatization is linked to substantially reduced health-related quality of life, help-seeking and service utilization among trauma survivors are very low. To date, there has not been available in Iceland a culturally attuned, self-reported measure on help-seeking barriers after trauma. This study aimed to translate and cross-culturally adapt the English version of Barriers to Help-Seeking for Trauma (BHS-TR) scale into the Icelandic language and context.

**Methods:**

The BHS-TR was culturally adapted following well-established and rigorous guidelines, including forward-backward translation, expert committee review, and pretesting through cognitive interviews. Two rounds of interviews with 17 female survivors of intimate partner violence were conducted using a think-aloud technique and verbal probing. Data were analyzed using qualitative content analysis, a combination of deductive and inductive approaches.

**Results:**

Issues with the BHS-TR that were uncovered in the study were classified into four categories related to general design, translation, cultural aspects, and post-trauma context. The trauma-specific issues emerged as a new category identified in this study and included concepts specific to trauma experiences. Therefore, modifications were of great importance—resulting in the scale becoming more trauma-informed. Revisions made to address identified issues improved the scale, and the process led to an Icelandic version, which appears to be semantically and conceptually equivalent to the original version; additionally, the results provided evidence of content validity.

**Conclusions:**

As a cognitive interview study, it adds to the growing cognitive interviewing methodology literature. Furthermore, the results provide essential insights into the self-report response process of trauma survivors, highlighting the significance of making health-related research instruments trauma-informed.

## Plain English summary

Trauma (being hurt a lot) can have big effects on people’s health and how they feel about life. However, very few people who are hurt by others look for help, even at places that have been set up to help them and others, like health clinics. Understanding how hard it can be for people who have been hurt by others to actually get the help they need can make it easier for us to help them sooner and in better ways. Before, in Iceland, there has been no easy way to study what stops people from looking for help after being hurt. In this study, we changed the English language Barriers to Help-Seeking for Trauma (BHS-TR) scale so it was a better fit for Icelandic people who have had trauma in their lives. We talked to 17 Icelandic women who had lived through violence at home. We wanted to know what they thought of the BHS-TR scale and how to make it more useful in Iceland. In general, they liked the scale but they had several ideas about how to make it better and how to make sure that taking the survey did not make the women feel bad about their lives. We made changes to the scale based on their ideas.

## Background

Much of the global population is exposed to traumatic events in their lifetime [1, 2]. Such events are defined as exposure to threatened death, serious injury, or sexual violence [3]. While most individuals exhibit resilience and the capacity to recover afterward [4–6], many survivors face lasting adverse effects, leading to trauma identified as a public health issue [7, 8]. Traumatization is linked to an increased risk of suffering from an array of mental and physical conditions associated with functional impairment and substantially reduced health-related quality of life (HRQoL) [9, 10], even years after exposure [11]. This suffering is particularly evident in cases of complex and interpersonal trauma [7, 12]. A recent review of the WHO world mental health surveys in 24 countries (*n* = 68,894) showed that of the 29 trauma types assessed, interpersonal traumas carried the highest posttraumatic stress disorder (PTSD) risk, and intimate partner violence (IPV) had the highest PTSD burden [5].

Despite these adverse effects of trauma on HRQoL, the estimates of help-seeking among traumatized individuals are very low, especially for formal sources of help, including the healthcare system [13–15]. Thus, a significant proportion of trauma survivors are not receiving the care they need. This finding is consistent with previous research that has shown that survivors are facing a range of barriers to health service utilization, including both system-level structural factors and individual attitudinal factors [16–18]. Further exploration of the barriers that trauma survivors face is essential to better understand the gap between the perceived need for help and service utilization, which can give us the critical information needed to increase help-seeking after traumatization. However, instruments measuring help-seeking barriers have mainly been developed in the specific context of physical or mental illness, and very few are trauma-specific [19–21].

The Barriers to Help-Seeking for Trauma (BHS-TR) scale was developed by Saint Arnault [17] based on the Barriers to Seeking Care scale used in the mental health supplement of the Ontario epidemiology study [22]. The original scale included 25 barriers to mental health service utilization. Besides Canada, these barriers have been examined in large population-based studies in the United States and the Netherlands and found to be relevant, despite differing healthcare systems in these countries [23]. For the BHS-TR, Saint Arnault added items related to help-seeking after traumatization, based on literature review and focus group interviews with gender-based violence (GBV) survivors from the United States and Ireland. The BHS-TR has been used to date in research with women from both primary-care sites and the general community [17, 24]. Moreover, the scale was found to be reliable, valid, and useful in understanding aspects of the barriers GBV trauma survivors experience on their help-seeking journey (Saint Arnault, D. M., & Ozaslan, Z. (under review). Understanding help-seeking barriers after gender-based violence: Validation of the barriers to help-seeking for trauma (BHS-TR) scale. *International Journal of Mental Health Nursing*.). BHS-TR is, to our knowledge, the first trauma-specific instrument that assesses barriers to help-seeking. As has been noted, the scale was developed from existing mental health barriers measure but adapted for survivorship, which can be related yet distinct. The rationale for choosing the BHS-TR was the need for a trauma survivor-centered help-seeking barriers instrument that can be used to identify and develop interventions to mitigate them and help revise services to address these barriers in Iceland.

Investigators are increasingly turning their attention to best practices for instrument translation and cultural adaption [25–27]. It is recognized that if measures are to be used across cultures, the items must not merely be translated well linguistically but also adapted culturally to maintain the content validity at a conceptual level [28]. The most appropriate way to collect data to support content validity is by conducting qualitative research involving communication with participants to capture their perspective on the measure [29]. Within the existing guidelines, there is furthermore a broad agreement on the purpose and necessity of pretesting the translated instrument with the target population [27, 28, 30].

Cognitive interviewing (CI), a psychologically oriented method for empirically studying how respondents mentally process and respond to survey questions [31], has emerged as an essential qualitative method for the pretesting and evaluation of self-report instruments. Its primary value is in providing information to uncover potential issues with questions and offer recommendations for improvements [32, 33]. However, since CI was derived from the cognitive aspects of survey methodology movement [34, 35], the method has been criticized in the context of cross-cultural research for lack of focus on sociocultural factors that can influence the survey response process, consistent with the understanding that cognitive processes do not operate within a black box but are shaped by persons’ lived experiences [33, 36].

Cross-cultural cognitive interviewing (CCCI) is a variant of standard cognitive testing that has increasingly been carried out in an effort to detect issues related to the translation of instruments and establish cultural equivalence [37]. This extension of CI, an already interdisciplinary paradigm, has incorporated perspectives from sociology and anthropology [32, 38], with an increased emphasis on how members of different cultural groups interpret specific questions and instruments in the context of their unique viewpoints [33, 37].

Currently, there is no self-reported measure on help-seeking barriers after trauma available in Iceland. This study was undertaken to translate the BHS-TR scale from the English version into the Icelandic language and to adapt it for use in the Icelandic context. Also, we aimed to pretest and qualitatively evaluate the content validity of the Icelandic version through cross-cultural cognitive interviews. This study is part of a larger international research being carried out by the Multicultural Study of Trauma Recovery (MiStory).^1^

## Methods

### Description of the instrument

The BHS-TR is a 34-item self-report instrument, measuring barriers to seeking help for trauma recovery [17]. Help-seeking is defined as “the experiences, expectations, and interpretations that interact with structural variables, as well as context, to influence behavior aimed at reducing suffering and promoting health ([24] , p. 163).” The scale includes structural barriers (e.g., lack of information, financial problems, and unavailability of care), intra- and interpersonal barriers (e.g., normalization, feeling they must solve it on their own, and shame), and trauma-specific barriers (e.g., feeling frozen, confused, or fearing the consequences of disclosure). Respondents answer on a Likert scale anchored at 1 (“Did not influence me”) to 4 (“Strongly influenced me”).

### Translation and adaptation process

**Fig. 1:**
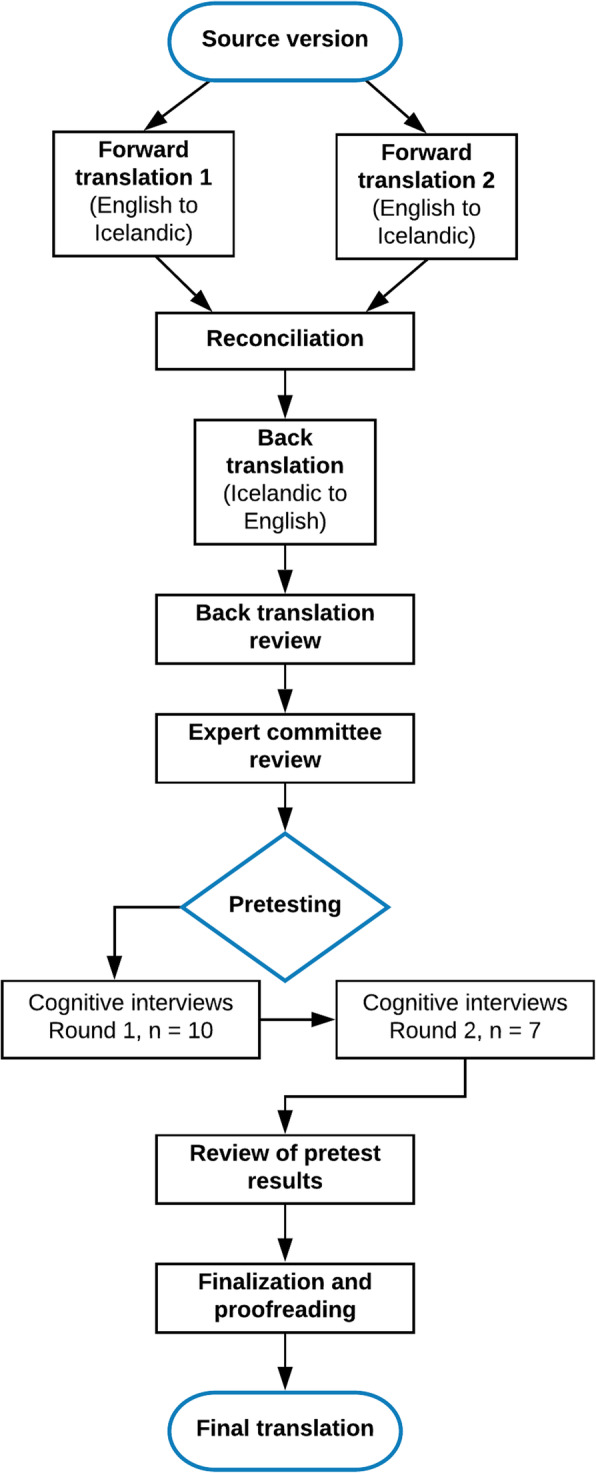
A flowchart of the cross-cultural adaptation process

The BHS-TR was translated and culturally adapted following international guidelines and principles of good practice [27, 28] (see Fig. 1). These guidelines have been widely used for translation and adaption of self-reported measures, providing a rigorous process designed to maximize the attainment of semantic and conceptual equivalence between the source and translated instruments. Steps of the process are described in detail in Table 1.

**Table 1:** Overview of the cross-cultural adaptation process, based on [27, 28]

Steps	Description of how each step was performed
Step 1. Preparation	The instrument developer (last author) permitted the process and agreed to be involved.
Step 2. Forward translations	Two separate forward translations were carried out individually by two native Icelandic speakers who were fluent in English, one a professional translator and the other a health professional; both also experienced researchers in their field. The focus was to avoid literal translation and capture the conceptual meaning of the items.
Step 3. Reconciliation	The first author, a native Icelandic speaker, met with the translators to synthesize the results of the translations. They compared and discussed the differences between the two translations. Ambiguities and discrepancies were examined to create a single forward version.
Step 4. Back translation	Back translation of the reconciled translation was performed by a native English speaker who was fluent in Icelandic. The back translator was an experienced researcher in the field of health sciences but had no prior knowledge of the instrument and had not seen the two forward translations.
Step 5. Back translation review	The first author reviewed the back translation against the source instrument to identify any discrepancies and ensure the conceptual equivalence of the translation. Clarification and recommendations were sought from the instrument developer (last author, a native English speaker and a skilled researcher).
Step 6. Expert committee review	A multidisciplinary committee reviewed all the versions of the instrument (steps 2, 3, and 4) and developed the pre-final version of the translation. The committee consisted of a methodologist and two health professionals (nurse and psychologist) experienced with working with survivors of violence. The committee’s role was to make recommendations to achieve semantic, idiomatic, experiential, and conceptual equivalence between the source and target instruments.
Step 7. Pretesting through cognitive interviews	Two rounds of cognitive interviews were conducted with participants drawn from the target population. The goal was to explore comprehensibility, interpretation, and cultural relevance of the pre-final version. The interview data were analyzed using qualitative content analysis.
Step 8. Review of pretest results	The results were reviewed, and desirable modifications identified. The pretesting, being of particular importance for cultural adaptation, is described in more detail in the sections below.
Step 9. Finalization and proofreading	Following agreement on adjustments, the translation was finalized. In this final quality control step, the translation was proofread by one of the forward translators, which checked for any remaining spelling or grammatical errors.
Step 10. Final report	Each step was thoroughly documented during the process, allowing tracking of all the decisions made. This article represents the final step of the process, where a report is written on the development of the Icelandic version of the scale.

#### Ethical considerations

Permission from the instrument developer to use, translate, and adapt the BHS-TR was obtained. The National Bioethics Committee in Iceland provided the ethical clearance to conduct the CI in the pretesting phase (VSNb2019060009/03.01), and the study was reported to the Data Protection Authority (19–119). Verbal and written informed consent was obtained before participation, and all participants received a list of local referral resources at the end of the interview. They were also offered post-interview support from a psychologist if difficult emotional reactions emerged during or after participation.

### Cognitive interviews

The Icelandic version of the BHS-TR was pretested using cognitive interviews. The theoretical perspective underlying this study was Tourangeau’s [34] four-stage cognitive model of survey response: (1) comprehension of the question, (2) retrieval from memory of relevant information, (3) judgment of needed information, and (4) responding to the question. Due to the cultural aspect, the pretesting was furthermore conducted following recommendations for CCCI [37], with a focus as well on sociocultural factors that can influence the response process.

#### Interview participants

Seventeen women took part in the interviews. The inclusion criteria were: Icelandic women (aged ≥18 years) who self-identified as having experienced IPV. For ethical reasons and the safety of the women, they had to have been out of the abusive relationship for at least a year. Participants were recruited from centers and services for survivors of violence in North and South Iceland, using purposive sampling. The years in the abusive relationship ranged from one to eighteen, and most had been in the relationship over six years. The years out of the abusive relationship at the time of the interviews varied from two to twelve, on average, five years. Most of the women (13) had sought help from informal sources (family and friends) at the time of leaving the abuse, but few (6) had done so at later stages in the help-seeking journey when trying to recover from the trauma. The women had all sought formal help for their traumatic experience, hence the recruitment strategy. Nevertheless, only nine of them had sought help from the healthcare system, even though all had been faced with adverse, emotional, physical, and social health effects of the violence. During recruitment, attempts were made to select a diversity of individuals within the target population, as recommended for CI [32]. Table 2 describes the participants’ socio-demographic characteristics.

**Table 2:** Overview of the participants’ demographics

Characteristics	*N* = 17
Age	
18–29	4
30–39	7
40–49	4
50+	2
Icelandic language proficiency	
Native speaker	16
Fluent speaker	1
Education completed	
Less than high school	3
Junior college	5
University degree	9
Employment status	
Employed	12
Unemployed	5
Number of children	
None	5
One or two	9
Three or more	3

#### Procedures

Two iterative rounds of in-person CI were conducted, a total of 17 interviews. The interviews took place between August and October 2019, in a location chosen by participants, either at their homes or in a secure meeting room. The first author conducted all the interviews, which lasted on average for an hour. Think-aloud technique and verbal probing were used in conjunction to elicit participants’ interpretive process. The probing was done concurrently, using a combination of scripted and spontaneous probes (see Table 3). All interviews were audio-recorded with participants’ permission, and written notes were taken by the interviewer, documenting non-verbal cues. Round one consisted of interviews with 10 participants. Items identified as problematic during the analysis in the first round were revised and then further tested in round two with seven additional participants. In both rounds, all items on the scale were examined, in addition to obtaining feedback on BHS-TR relevance and cultural attunement in the Icelandic help-seeking context.

**Table 3:** Cognitive probes based on Tourangeau [34] and Willis [37]

Category of probes	Example
Comprehension probe	What do you understand by …?
Interpretation probe	What does the term … mean to you?
Paraphrasing probe	Can you tell me in your own words what this item is asking?
Process probe	How did you arrive at that answer?
Recall probe	How do you remember that you …?
Elaborative probe	Tell me more, why do you say that?
Sensitivity probe	Is it all right to ask about this, or do you think that this item is inappropriate?
Evaluative probe	Do you feel this item is easy or difficult to answer?

#### Data analysis

After each interview, the audio recordings were reviewed and transcribed. Next, detailed summaries were prepared, using the transcripts and written notes from the interviews. These interview summaries were used as the main source for the analysis. The first author was the primary analyst, responsible for preparing the interview summaries, coding, and categorization, with a thorough follow-up on the whole process from the second author.

**Fig. 2:**
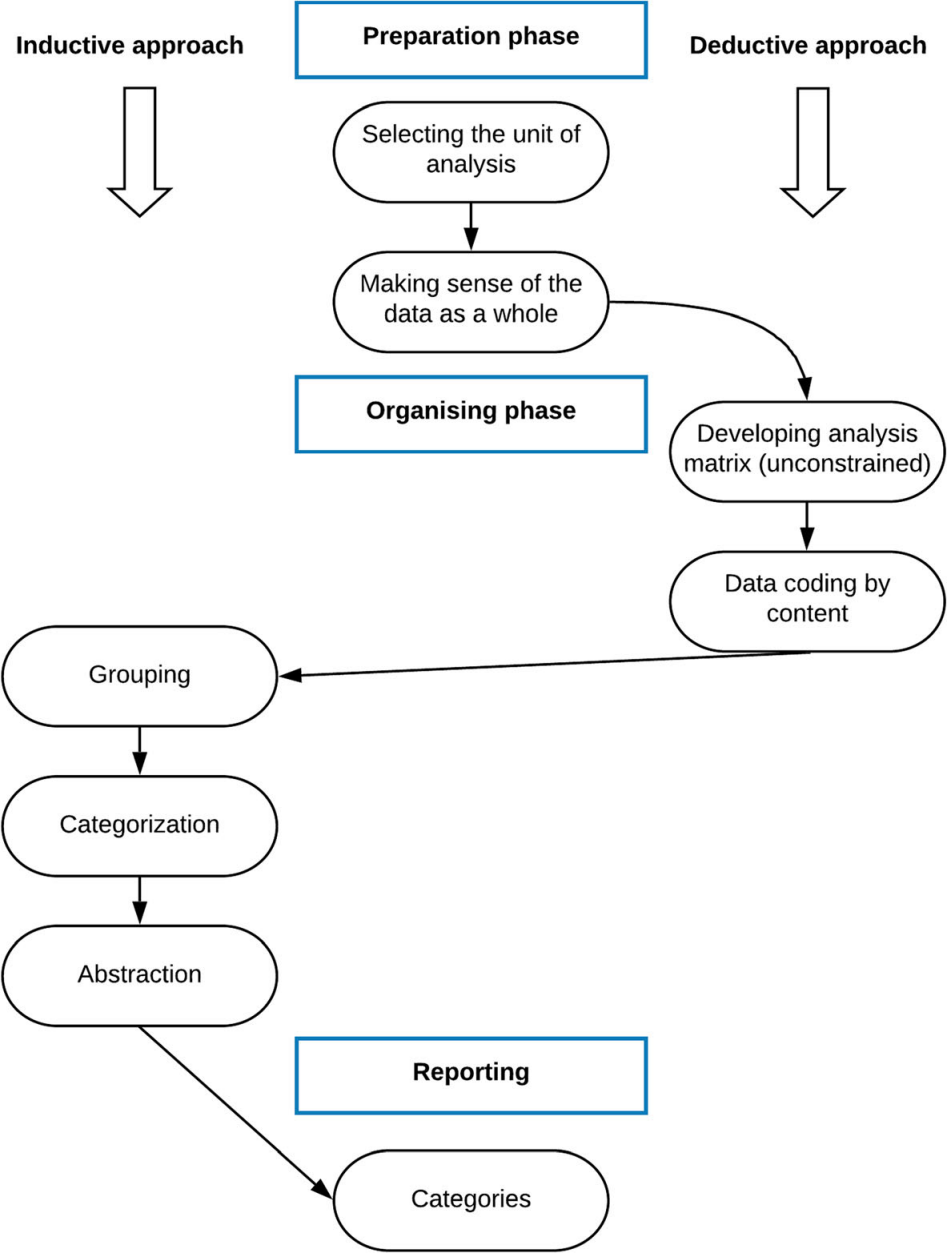
Overview of the analysis process, modified from [39], used with permission

A qualitative content analysis (QCA) was performed based on the procedures described by Elo and Kyngäs [39], focusing both on manifest and latent content. The main focus was to identify issues that participants had with the scale and classify them into meaningful categories. The analysis utilized a combined deductive and inductive approach, using an unconstrained matrix to code the data (see Fig. 2). The matrix was mainly based on categories frequently reported in the literature; general design issues, translation issues, and cultural issues [40–42], but since it was unconstrained, there was a possibility of creating different categories within its bounds [39]. In addition to the more familiar categories identified using the deductive approach, trauma-specific issues emerged as a unique category in this study, using steps of the inductive approach (grouping, categorization, and abstraction). The sub-categories within each of the four categories were also created following those steps. The analysis process was performed after each round, and afterward, the results were examined together before making final decisions about revisions. All authors were involved in the dialogue and the careful determination of revisions made. An audit trail was maintained for scientific rigor.

## Results

### Translation and adaptation process

The two forward translations were concordant on most items and considered linguistically equivalent in terms of their meaning. Minor differences were in wording, and for the reconciliation version, preference was given to less complicated and semantically equivalent words. Review of the back translation suggested semantic and conceptual equivalence with the source version as most items were nearly identical. Translation discrepancy identified in the review was regarding the item “I had distance or transportation problems,” which was translated to “I had problems going there” and hence became broader in the translation process than intended. Therefore, the translation of the item was made more specific, using the Icelandic words for “distance” and “transportation.”

The expert committee provided remarks on several items, mainly involving minor grammatical changes and suggestions for different wording, as special attention was given to producing a translation that would be easily understood by the target population. Other challenges reported by the committee involved the cultural relevance of items, for instance, concerning health coverage. Due to different healthcare and insurance systems, the committee recommended modifications. The same was true for items regarding race and ethnicity, which the committee thought might need to be adapted better to the Icelandic context. These items were kept unchanged for further assessment in the pretesting.

### Cognitive interviews

The BHS-TR was well-received overall, and the women stated that the items described their experience with barriers to seeking help after trauma for the most part. However, the participants questioned the relevance of a few items, which were the same items of concern by the expert committee. The women found the layout of the scale to be clear, the response options appropriate, and most items understandable, and the CI confirmed that they grasped the intended meaning. Furthermore, the women talked about the importance and value of this measure concerning their own challenges while trying to seek and seeking help. In the interviews, most of them had a hard time or felt uncomfortable with the think-aloud technique, whereas the probes were generally adequate.

Several issues, primarily on item-level, were uncovered in the interviews using the deductive and inductive QCA. The issues were classified into the four following categories: (1) *General design issues:* Problems with the design or layout of the original scale, (2) *Translation issues:* Problems where the wording of a translated item altered the intent of the source item, (3) *Cultural issues:* Problems where differences in social structures, norms, or values made it challenging to convey items to the Icelandic context, and (4) *Trauma-specific issues:* Problems where participants had trouble responding or were sensitive to the wording of items due to their lived experiences of trauma.

More detailed examples of the identified issues are presented in the following sections, as well as the revisions that were made as a result of both pretesting rounds. Quotations from participants that best represent and support each identified issue were chosen (see Tables 4, 5, 6, and 7). To allow for cross-cultural comparison, we were partially restricted in making some modifications to resolve these issues. Revisions that were made, based on the first pretesting round, reduced the number of issues identified in the later round, nearly eliminating items with problematic translations and enhancing equivalence between the translated and source versions. In the second round, similar insights were repeated, and no new significant problems emerged. Thus, it was decided that no subsequent rounds were required.

**Table 4:** Examples of general design issues identified

Issues	Description	Meaning unit	Revisions
Unclear scale instructions	Many (13 of 17) participants thought the scale instructions were unclear regarding the time of being faced with these barriers and how to answer if they had, at some point, sought help after their traumatic experience.	“Like what time are we talking about? And what if I did seek help at some point, should I still answer the scale?”	The specific time frame of asking about the last 12 months was added. Clearly stating that if people felt that they needed help during this specific time period but did not seek it, they are asked to answer the scale, even though they had sought or received help in the past.
Response options are forgotten	Several (6 of 17) participants forgot what the response options stood for while answering the scale.	“Halfway through, I forgot what the answer possibilities stood for, you should maybe have them visible at each item, so people do not have to scroll up and down.”	Description of the response options was made to appear more frequently on the scale.
Double-barreled items	Items with the conjunctions “or” and “and” were thought to be confusing and hard to answer by a few participants (3 of 17).	“I do not like these statements with “or” in them, you know asking about two things … it is a little confusing to answer. Also, if I have experienced only the other, how do you know which one I meant?”	None at this point, since fully addressing the issue would require significant modification to the scale.
Repetition	Some participants (5 of 17) thought that the scale included repetitive items.	“I just answered this … I know you are probably going to be looking at if I answered these questions in the same manner, but that is just really annoying.”	No need to adjust, as the BHS-TR is a multidimensional measure, and repetition in this context (and for that purpose) is consistent with good study design.

**Table 5:** Examples of translation issues identified

Issues	Description	Meaning unit	Revisions
Problematic translation	In the item “I thought the problem would probably go away by itself,” the phrasal verb “go away” was translated to “disappear” in Icelandic (9 of 17 participants).	“Disappear is not really appropriate for this kind of problem. Sure, I thought things would get better by itself, but never just poof and gone.”	The translation was changed to “I thought the problem would probably get better by itself.”
Problematic translation	In the item “I was ashamed or embarrassed,” the word “embarrassed” was translated as “awkward” in Icelandic, which was unsuitable in this context (12 of 17 participants).	“Awkward? I was racked with shame not blushing … using this word is not okay in my opinion.”	Since being ashamed and embarrassed have similar meanings in Icelandic, the adapted item became “I was ashamed.”
Problematic translation	In the item “I couldn’t get time away from work or family responsibilities,” the Icelandic meaning of the word “responsibilities” is more similar to the English word “obligations” (8 of 17 participants).	“Is taking care of my family supposed to sound so negative?”	The word “responsibilities” was cut from the item, changing the translation to “I couldn’t get time away from work or my family.”
Problematic translation	In the item “I thought getting help would take too much time or was inconvenient,” the word “inconvenient” was translated to “impractical,” which was missing the aspects of causing trouble or discomfort (6 of 17 participants).	“I would put that this did not influence me because you don’t think about if it is going to be practical or not … is this really the best word to use?”	The Icelandic word “troublesome” was used instead.

**Table 6:** Examples of cultural issues identified

Issues	Description	Meaning unit	Revisions
Connotations and cultural idioms	In the item “I was afraid I couldn’t clearly express my needs,” the word “needs” was considered by many participants (11 of 17) to be strange in this context. They thought Icelanders would instead use sayings like “how I felt” or “what was wrong.”	“What do you mean by my needs? You know, I know what it literally means, but I would never sit down with my doctor or psychologist and say I need this and that.”	Despite good suggestions from participants, those changes would have altered the literal meaning of the item. The wording was nevertheless changed from “my needs” to “what I needed.”
Different healthcare system	All (17 of 17) participants commented on the item, “My health coverage wouldn’t cover the type of treatment I needed.” Some thought the item was not appropriate in the Icelandic context, while others thought it was relevant regarding some mental health services.	“This sounds very American, we don’t say our or my health insurance, everybody is insured … the system here is so different, and it covers a lot of care. Although not all mental healthcare is government-subsidized, so that affects trauma survivors in Iceland.”	The adapted version of the item became “The available health insurance wouldn’t cover the type of treatment I needed.”
Historically homogeneous nation	Some (8 of 17) participants wondered if the following item, “I felt that my culture, ethnic background or specific situation would not be understood,” should be on the Icelandic version of the scale. However, they often stated that if immigrants were to answer the scale, then this item might be relevant.	“Is this appropriate in Iceland? I don’t think that culture or ethnic background is a problem for most Icelanders … then again, we are becoming more diverse … still, I feel like there is a lot in your background besides ethnicity that you can be scared of people not understanding, so you don’t seek help.”	The item was adapted to the Icelandic context by changing it to “I felt that my culture, background or specific situation would not be understood.”
Historically homogeneous nation	In the item “I felt that there would be prejudice, racism, or discrimination against me,” several (9 of 17) participants focused only on the word “racism” and thought that prejudice or discrimination had to be related to their race.	“This focus on race is not very Icelandic, so yeah, I would answer that this did not affect me because you know of course I thought there would be prejudice, but not about my race or you know racism.”	The adapted version of the item became “I felt that there would be prejudice or discrimination against me,” which could include racism, among other things.

**Table 7:** Examples of trauma-specific issues identified

Issues	Description	Meaning unit	Revisions
Post-trauma context	Difficulties deciding how to answer due to the complex nature of the trauma and a multistep help-seeking journey was a common problem (14 of 17 participants).	“Some of these barriers are also really descriptive when you are trying to leave an abusive relationship, so my mind always went there. When I think about seeking help for my PTSD or you know everything after … there can be different barriers, so I would answer differently.”	The scale was designed to measure barriers to seeking help for trauma recovery, which was included in the scale instructions. However, this issue made it apparent that it needed to be more clearly defined. It furthermore supported the change of adding the specific time frame of asking about the last 12 months.
Post-trauma context	Some (8 of 17) participants felt that the following item “Professionals from my own cultural or ethnic group were not available” should rather be about the gender of professionals than about their culture or ethnicity. Hence, this problem was also classified as a cultural issue, but the primary meaning was related to the post-IPV context (also relevant to other types of interpersonal trauma).	“I do not relate and actually it doesn’t matter to me what their ethnicity is or culture, cultural … uhm … I don’t even know what my cultural group is, you know we don’t talk like this, anyway … what was really a barrier for me was being forced to have a health professional of the same gender as my perpetrator.”	The adapted version of the item became “Suitable professionals were not available,” which could include the cultural and ethnic background as well as gender.
Sensitive to wording	The wording “your decisions not to seek help” in the scale instructions was thought to be offensive by many (10 of 17) participants.	“It is not this simple, I did not sit down and made this thought out decision to not get the help that I needed.”	The instructions were rephrased using the word “reasons” instead of “decisions.”
Victim- blaming wording	Several (7 of 17) participants thought some of the items had victim-blaming wording.	“My problems, my situation, my my my … you are taking all of the responsibility from the perpetrator and putting it all on the survivor like it is my fault.”	Revisions involved changing the word “my” to “the” in the problematic sentences that reflect negative aspects (such as problem and situation) while maintaining “my” in those sentences that reflect respondent autonomy (such as reasons, feelings, or actions).
Sensitive to word order	Few (5 of 17) participants were sensitive to the order of words in the following item “I was worried that if others discover my health problems or my situation, I could lose housing, security, or my children.”	“I would never put housing and my security before my children … so I would not want to answer this.”	The order was changed to “my children, security, or housing.”

#### General design issues

The general design issues were divided into four sub-categories, involving unclear scale instructions, forgetting response options, double-barreled items, and repetition of items (see Table 4). Overall, we detected relatively few general design issues. However, these were the issues that we were most restricted in making modifications. The lack of clarity of the scale instructions, being of particular importance, and also related to the post-trauma context issues (see Table 7) regarding the women’s multistep help-seeking journey was addressed by adding the specific time frame of asking about the last 12 months.

#### Translation issues

There was only one sub-category of the translation issues identified, problematic translation, which was when the Icelandic words did not convey intended constructs or meaning (see Table 5). This category had issues that were most easily resolved, and modifications mainly involved choosing alternative words. Very few problematic translations were identified in the second round of CI.

#### Cultural issues

The cultural issues were divided into three sub-categories, all related to the appropriateness of items in the Icelandic help-seeking context (see Table 6). Firstly, there were few issues regarding connotations, cultural idioms, or the use of phrases in the speech of Icelanders. Secondly, while going through the scale, the women talked about the difference between healthcare in Iceland versus the United States. These cultural differences were especially regarding items about cost and individual health coverage, which they did not relate to since Iceland has a universal healthcare system that is primarily paid for by taxes and under which all legal residents are covered. Thirdly, some of the women viewed Iceland as being a relatively homogeneous country, and therefore, found items about race and ethnic background to be culturally inappropriate. Revisions mainly involved making adaptations based on the women’s input, while still maintaining cross-cultural comparability.

#### Trauma-specific issues

The trauma-specific issues were divided into four sub-categories, where the underlying thread was the unique post-trauma context and how it impacted the participants’ viewpoints (see Table 7). The women had difficulty answering some items due to the complex and interpersonal nature of their trauma; some described general difficulties responding to the measure because the specific barriers to seeking help for trauma recovery reminded them of parallel barriers they had experienced over time while seeking to live through and survive their own traumatic experiences. Another aspect of these issues was the participants’ vulnerability, where they were sensitive to the wording of items, the choice of words, and even the order of words. This category of issues became apparent and most associated with participants not wanting to answer, and ultimately skipping items. It also became clear that few items on the scale were triggering negative feelings for some women. Therefore, modifications were of great importance, and these issues were resolved with clarification or rewording items, making the instrument more trauma-informed as a result.

## Discussion

This study described the translation and cultural adaption process of the Icelandic version of the BHS-TR, including cognitive interviews with survivors of IPV. To the best of our knowledge, this is the first Icelandic trauma-specific instrument that assesses barriers to help-seeking. The process led to a version that is semantically and conceptually equivalent to the original English version; additionally, the results provided evidence of content validity.

The first steps of the translation went without significant problems, which might have been facilitated due to English and Icelandic both being Germanic languages. Interestingly, only one problematic translation issue emerged during the back translation step. However, several translation issues were independently identified with both the expert committee and the CI, making us question if we could have forgone the back translation step and potentially adding to the growing body of research indicating the shortcomings of back translation as a quality testing tool [43, 44]. Still, it could be that the efficacy of the back translation was limited by the nature of our study, as back translations can be particularly valuable in cases where multiple target languages are being developed simultaneously, and can therefore be analyzed as a group, and less so when only one target language is being developed.

The pretesting through CI was a valuable step, allowing us to gain insight into participants’ perspectives and interpretations of the scale that helped us to identify issues and improve the Icelandic version before field testing. CI has been used successfully in many areas of healthcare research to develop and culturally adapt instruments [45, 46]. The theoretical perspective underlying the interviews in this study was from traditional CI [34] with influences from CCCI [37]. Hence, we not only focused on what is presumably happening in the “black box” of the mind [47] but also how these cognitive processes are tied to the sociocultural context, which was essential to successfully adapt the instrument to the lived reality of Icelandic trauma survivors. During the interviews, the women found it difficult to “think out loud,” which is in line with other studies where difficulties with this technique across diverse linguistic and cultural groups have been reported [48–50]. On the other hand, the concurrent probing functioned well and helped the women elicit their interpretive and meaning process.

Results from the interviews showed that the women found the Icelandic version of the BHS-TR in general clear, understandable, and relevant. Moreover, we identified relatively few general design and translation issues, which might have resulted from the rigorous steps in the translation and adaptation process taken before the pretesting.

Iceland, as a high-income Western country, shares numerous cultural characteristics with Canada and the United States, where the scale was developed, including a strong sense of individualism and a great emphasis on independence and self-help [51], which can influence help-seeking and service utilization. Yet, we detected a few cultural relevance issues, mainly involving Iceland being less ethnically and culturally diverse than those two countries. Another observed distinction was related to different types of welfare states, a critical factor in the formal help-seeking context. A similar issue was reported in a study adapting a U.S. developed healthcare-related measure for use in another Nordic country [46].

The trauma-specific issues category, unlike the others, was not built on common categories identified in previous research. These were nevertheless the issues that most resulted in participants skipping items, which is noteworthy since one of the aims of CI is to ensure high response rates from a sample of the target population in the field testing [45]. This category is a valuable finding for those undertaking studies with trauma survivors or other vulnerable populations and highlights the importance of making research instruments trauma-informed.

Importance was given to the analysis process of the interviews as it has been identified as the most undeveloped area of CI methodology; researchers rarely describe how they moved from data collection to the production of results and revisions [47]. QCA was believed to be the most suitable method since the aim was to identify any issues participants had with the scale, displayed as manifest or latent content, and to classify these issues into smaller content categories. This method has been used effectively in other CI studies [40, 42]. One challenge in using a deductive approach, however, is deciding how to treat meaningful left-over data [52]. The decision to use an unconstrained matrix gave us the flexibility to create the new category following the principles of an inductive approach [39], making the left-over data become an essential contribution. The combination of deductive and inductive approaches within QCA has recently been described as an abductive approach [52]. To enhance the study’s trustworthiness, we undertook a joint collaborative analytic process, along with maintaining a transparent audit trail, showing the analytic steps, and linking the revisions to the data as recommended when using QCA [39, 53].

The findings in this study are subject to some limitations—including a relatively small sample size of 17 participants. However, traditionally CI studies include few participants but strive to conduct interviews with a variety of individuals [32, 54]. The target population in this study was Icelandic female IPV survivors, and we believe that our sample contains important characteristics of that population. We did not select participants according to racial categorization. All the women would, nevertheless, be socially classified as “white,” and even though that mirrors most Icelandic women, we are aware of this limitation, particularly regarding the women’s view on the relevance of items related to ethnic background and race, and the likelihood that minority populations are likely to experience unique barriers to help-seeking and care which is the primary focus of this scale. With globalization and growing migration, the Icelandic nation is becoming more diverse [55], and it may at some future date be desirable to further culturally review and adapt this Icelandic scale to be of maximum utility to populations not well-represented in our sample.

The data collection was stopped after two rounds of interviews when saturation was reached. According to Willis [54], an instrument could, in one sense, be tested forever and still have issues. The BHS-TR was tested until several major issues had been detected and adequately addressed. This study did not include source-language interviews with English-speaking survivors. An emerging consensus within the CCCI literature is that source-language testing should be done in parallel with the translation assessment, to establish whether the problems identified are truly general, rather than specific to the target version [37, 42]. Presumably, this approach would have provided important data, especially for dealing with general design issues as well as allowing for decentering, which has been defined as the process of modifying the source instrument when necessary along with the translated version [56].

After an instrument has been translated and adapted, additional testing of the psychometric properties is highly recommended [28]. Although the interviews provided evidence of content validity and valuable insight into the women’s interpretations of the scale, it does not address the reliability, item response patterns, or construct validity. Further testing of the Icelandic BHS-TR is therefore needed. As mentioned earlier, the pretesting in this study was limited to female IPV survivors seeking formal sources of help to meet healthcare needs. Based on the literature reviewed above, indicating low rates of help-seeking from any source despite a diminished HRQoL, it is crucial to test the scale with trauma survivors not seeking help. Future research should as well include more diverse populations regarding gender, ethnic origin, and the types of trauma experienced.

## Conclusions

In this study, we translated and adapted the BHS-TR into the Icelandic language and context. The process followed a series of ten rigorous steps, where the expert committee review and the cognitive interviews were particularly useful steps. The study adds to the growing CI methodology literature, where the emphasis was put on reporting of the analysis. The results provide essential insights into the self-report response process of trauma survivors, highlighting the significance of making health-related research instruments trauma-informed. Revisions made to the scale improved it, and the process resulted in an Icelandic version, which appears to be semantically and conceptually equivalent to the source version, as well as linguistically valid. Evaluations of the scale’s psychometric properties are, however, recommended. The availability of a valid and reliable trauma-informed measure of help-seeking barriers has a value for health research, practice, and policy—by providing information that can guide the development of evidence-based interventions to break down barriers and facilitate help-seeking after trauma.

## Data Availability

The datasets generated and analyzed during the current study are not publicly available due to them containing information that could compromise participants’ privacy and confidentiality but may be made available from the corresponding author on reasonable request.

## Abbreviations

HRQoL: Health-related quality of life
PTSD: Posttraumatic stress disorder
IPV: Intimate partner violence
BHS-TR: Barriers to help-seeking for trauma scale
GBV: Gender-based violence
CI: Cognitive interviewing
CCCI: Cross-cultural cognitive interviewing
QCA: Qualitative content analysis

## Footnotes

1 The MiStory network (https://mistory-traumarecovery.org/) is a research collaborative currently working in 13 countries around the world to use safe and trauma-informed methods that illuminate the interactions among cultural context, the self, gender, and trauma recovery.
